# New Appliances and Things Medical

**Published:** 1908-08-15

**Authors:** 


### NEW APPLIANCES AND THINGS MEDICAL.

[We shall be glad to receive at our Office, 28 & 29 Southampton Street, Strand, London, W.C., from the manufacturers, specimens of all new preparations and appliances which may be brought out from time to time.]

### NEW PHOSPHORUS COMPOUNDS.

*Leciplasma.*—This is a light yellow powder, with a pleasant taste. It is prepared from the central nervous system and from egg-yolk, and contains lecithin 5 per cent., albumin, iron, carbohydrates, and salts. It may be taken pure or dissolved in warm water, milk, tea, soup, etc. One or more teaspoonfuls may be ordered thrice daily for adults, and smaller amounts for children. It may be given for all conditions in which lecithin or one of the organic forms of phosphorus is usually prescribed, and especially in exhausted conditions of the nervous system, and in anæmia and chlorosis. It is prepared by Lüttgen and Co., Berlin.

*Protylin* contains 2.6 per cent. of phosphorus combined with albumin; no heat is used in the manufacture, and the product keeps well. It is in the form of tablets, three or four of which (or possibly more) may be taken daily. It has been employed with good results to improve the appetite and increase the absorptive powers in anæmia, tuberculous adenitis, rickets, and various nervous and asthenic conditions. It appears to be very useful in children's diseases, as it is not unpleasant to take, and gives rise to no irritation in the stomach. It is prepared by Hoffmann, La Roche, Baden.

*Visnit* is a food preparation derived from milk, eggs, hæmoglobin, and cereals. It is said to contain no "extractives" or purin bodies. It is in the form of a fine yellowish powder with a faint, not unpleasing, taste. It may be used for months without exciting distaste or repugnance on the part of the patient. Analysis shows that it contains 7.14 per cent. of water, and 80.14 per cent. of nitrogeneous material, of which 1.85 per cent. is hæmoglobin. The ethereal extract amounts to 3.26 per cent., of which .24 per cent. is lecithin, and the salts (1.34 per cent.), which are said to be in an organic combination, contain .53 grams of phosphoric anhydride. The carbohydrates amount to 15.26 per cent., about one-third of which is in soluble form. A teaspoonful either dry or mixed with water or other fluid may be given thrice daily to adults, and half the amount to children. The indications are malnutrition and nervous exhaustion from any cause. The manufacturers are Hornvitz's Chemical Institute, Berlin.

